# Investigating and Summarizing Information Resources Related to the Clinical Presentation and Diagnosis of Cutaneous Manifestations of Infectious Diseases in Patients With Skin of Color

**DOI:** 10.1093/ofid/ofad692

**Published:** 2023-12-29

**Authors:** Dorothea McGowan, Anosh Kermani, John Sheagren

**Affiliations:** Infectious Disease, College of Human Medicine, Michigan State University, Grand Rapids, MI, USA; Infectious Disease, College of Human Medicine, Michigan State University, Grand Rapids, MI, USA; Infectious Disease, College of Human Medicine, Michigan State University, Grand Rapids, MI, USA

**Keywords:** cutaneous manifestations, dermatology, infectious diseases, skin of color

## Abstract

Patients with skin of color (SOC) present diseases differently in many circumstances, yet there is a lack of information regarding the presentation and diagnosis of cutaneous manifestations in such patients experiencing infectious diseases. Therefore, we conducted a scoping review to investigate and summarize information pertaining to the clinical presentation and diagnosis of cutaneous manifestations of infectious diseases in patients with SOC focusing on the following viral, bacterial, toxin-mediated, and infestation diseases and fungal infections: human immunodeficiency virus, shingles, impetigo, scarlet fever, Lyme disease, toxic shock syndrome, scabies, rickettsioses, and cutaneous fungal infections. This scoping review identified literature gaps regarding cutaneous manifestations of infectious diseases in patients with SOC such as a lack of visual examples and more precise descriptions of common infectious diseases. The lack of better-quality literature and educational resources describing cutaneous manifestations of infectious diseases in patients with SOC may contribute to care barriers; therefore, more research and collaboration are needed in the specialties of both infectious diseases and dermatology.

## Teaser

A scoping review of cutaneous manifestations of infectious diseases in patients with skin of color identified the lack of literature surrounding the topic. Increased research and collaboration are needed in the specialties of both infectious diseases and dermatology.

According to the 2018 National Hospital Ambulatory Medical Care Survey by Cairns et al [1], 7.2 million visits to physician offices in the United States were for infectious and parasitic diseases and 3.4 million emergency room visits had infectious and parasitic disease as the primary diagnosis. Diagnosing infectious disease is a routine part of these clinical settings and infectious diseases present differently in skin of color (SOC), which generally refers to a spectrum of complexion in a wide range of ethnic groups with varying susceptibility to ultraviolet skin damage, typically Fitzpatrick type IV–VI [2]. For example, a recent retrospective cohort study by Fawzy et al [3] regarding coronavirus disease 2019 (COVID-19) found that oxygen saturation was likely to be overestimated by optical pulse oximetry in people of color. When the authors compared the accuracy of optically driven pulse oximetry monitoring with arterial oxygen saturation of White patients to patients of racial or ethnic minorities, they found that one-third of patients of the racial or ethnic minority group had at least 1 unidentified episode of hypoxia, whereas just one-fifth of White patients had at least 1 episode of hypoxia. This overestimation of patient arterial oxygen saturation led to delayed eligibility to receive COVID-19 therapy in Black and Hispanic patients. This emphasizes the need for diagnostics that consider skin pigmentation [3].

Bhavani et al [4] compared temporal (using an infrared thermometer) and oral thermometer measurements in Black patients to those of White patients, concluding that there was significant measurement agreement between temporal and oral thermometers in White patients and significant measurement discrepancies in Black patients. This discrepancy suggests that infrared thermometers were inaccurate in Black patients.

Moreover, Black patients measured by the temporal method had 0.54 odds of meeting fever criteria compared to those measured orally. Such a discrepancy would lead to a delayed diagnosis of fever, causing delayed medical treatment in Black patients. Thus, patients with SOC present both disease and indicators of disease differently in many circumstances; yet, information is lacking regarding the presentation and diagnosis of cutaneous manifestations, specifically of those in patients with SOC who are experiencing infectious diseases [4].

Many studies have found that experiences involving patients with SOC are scarce in medical education, which may lead to the misdiagnosis of diseases in dark-skinned populations [5–8]. Because patients with SOC are less represented in medical textbooks, they may experience a delay in diagnosis of infectious diseases such as Lyme disease, as they require the presence of healthcare providers who can properly evaluate skin color for an accurate assessment/diagnosis [9–11]. A delay in diagnosis of Lyme disease due to failure to recognize cutaneous findings in dark skin leads to more severe complications such as arthritis and facial palsy [9, 12, 13].

Clearly, descriptions of cutaneous manifestations of infectious diseases in patients with SOC need increased emphasis. In this review, we summarize information resources pertaining to clinical presentation and diagnosis of cutaneous manifestations of infectious diseases in such patients. We focus on the following infectious diseases: human immunodeficiency virus (HIV), shingles, impetigo, scarlet fever, Lyme disease, toxic shock syndrome (TSS), scabies, rickettsioses, and cutaneous fungal infections. This review emphasizes the importance of taking skin color into account when diagnosing and properly treating a variety of infectious diseases occurring in patients with SOC. Furthermore, more robust studies need to be performed on recognizing and treating a variety of infectious diseases in such patients. There is a need for more educational experience regarding infectious disease presentation in SOC.

## METHODS

We examined the scope of published literature regarding the cutaneous manifestations of infectious diseases in patients with SOC following the Preferred Reporting Items for Systematic Reviews and Meta-Analyses Extension for Scoping Reviews (PRISMA-ScR) guidelines to identify knowledge and research gaps (the checklist is shown in the Supplementary Materials) [14]. We also referred to the Joanna Briggs Institute Reviewers Manual for guidance, in which the checklist provided in Chapter 11 allowed us to ensure that our scoping review was organized according to standards [15]. Two reviewers (D. M. and A. K.) screened titles and abstracts for eligibility, then evaluated the full text of selected articles to assess for relevance. Disagreements in article eligibility were discussed between the 2 reviewers.

In our scoping review, the databases PubMed and ScienceDirect were used to acquire only research and review articles; moreover, only resources provided in English were considered in the literature search. Articles were reviewed to see if they addressed cutaneous manifestations of infectious diseases in patients with SOC as a key topic. Only articles after the year 1987 were included. Research and review articles were identified using subject headings and keyword combinations. For inclusion, articles chosen needed to include information regarding cutaneous manifestations of infectious diseases in patients with SOC. Articles were excluded if they contained no information regarding an infectious disease–specific term in patients with SOC or if they included <2 sentences regarding infectious diseases and cutaneous manifestations in patients with SOC. We concluded our search in October 2023. Duplicates were also removed. The specific search terms used for the search are provided in the Supplementary Materials with headings pertaining to each specific section. The authors screened and evaluated the eligibility of the research and review articles according to the criteria outlined above.

## RESULTS AND DISCUSSION

The initial search yielded a total of 2894 articles. Of the original 2894 articles, 2792 were not related to the fields of infectious diseases and dermatology and were therefore excluded, leaving 99 full-text articles. Duplicates were then removed, yielding 92 full-text articles, which were further accessed for eligibility by excluding those that contained only 1 sentence pertaining to the topic of skin manifestations of infectious diseases in patients with SOC. After all exclusion criteria were applied, a total of 21 articles were included (Supplementary Figure 1).

Our review clearly demonstrates the paucity of literature describing infectious diseases manifestations in patients with SOC. We found that many articles contained only a few sentences on the topic. The articles ranged in dates from 1987 to 2023. The search terms that yielded the most results included: skin manifestations of infectious diseases in dark skin; infectious diseases in patients with “dark skin”; and HIV in “pigmented skin.”

HIV had the highest representation of infectious diseases cutaneous manifestations in patients with SOC, with 6 of the 21 articles pertaining to the discussion of HIV in patients with SOC [16–21]. Of the articles regarding HIV, most related to skin manifestations such as increased risk of opportunistic infection, photosensitivity, and treatment challenges [16, 17, 20, 21]

Only 1 result looked at education resources, specifically a study using perceptual and adaptive learning modules to increase skin assessment in patients with SOC [22]. The search yielded no eligible results for the topics of impetigo, scarlet fever, or scabies and therefore they are not discussed here. Although we did not search for the specific term “COVID-19,” many articles regarding the topic were found in the general infectious disease search term combination and therefore it is included below. The search terms that yielded results are discussed below and organized by pathogen class. A brief summarization of the included articles is provided in Supplementary Table 1.

### Viral Diseases

#### COVID-19

The significance of recognizing and understanding COVID-19–related dermatological manifestations in patients with SOC is underscored by recent research. One publication [23] states that having photos of SOC patients with COVID-19 dermatological manifestations is “essential in helping not only patients, but providers as well identify what may be early signs of infection.” However, such a statement only brings one's attention to the importance of early recognition, diagnosis, and treatment of COVID-19 in SOC patients potentially to reduce healthcare disparities. Two studies [23, 24] summarized that understanding the cutaneous manifestations associated with COVID-19 in SOC is crucial for healthcare practitioners as they systematically assess patients for potential signs and symptoms of the virus; moreover, it is imperative for healthcare professionals to be adept at identifying the skin-related manifestations of COVID-19 and understanding how they may manifest in a clinical setting [24].

#### HIV

People with HIV frequently experience skin infections from opportunistic infections, and skin infections are common in many HIV infection stages [16, 20]. As mentioned above, HIV had the highest representation of search results; however only 6 articles were found to be eligible. HIV-associated infections in darker pigmented skin can have various manifestations including hypopigmentation, hyperpigmentation, and scarring [16, 20]. HIV patients with pigmented skin encounter problems with diagnosis and treatment; moreover, due to dyspigmentation caused by conditions such as dermatophyte infections, early diagnosis is important to prevent disfigurement [20].

#### Shingles

One article regarding shingles was found, which included 2 sentences about the healing challenges from varicella skin lesions such as keloids, pitting, and hypopigmentation in patients with dark skin [25].

### Bacterial Diseases

#### Lyme Disease

Historically, one might entirely miss the diagnosis of Lyme disease in SOC by missing the important finding of erythema migrans, which is “less apparent and more difficult to diagnose in patients with SOC” [9, 26, 27]. One study found that within their sample of 6171 White Medicare patients and 167 Black Medicare patients, neurological manifestations of Lyme disease were found in about 34% of Black patients and only 9% of White patients [10]. The differences in neurological manifestations may well have been related to the failure to recognize the dermatological manifestations in Black patients, which led to delayed diagnosis and consequently disseminated disease [10]. Another study suggested that delayed diagnosis might have been more associated with increased length of stay and costs for SOC patients with Lyme disease [27]. A more recent article briefly touched upon the hypothesis that a erythema migrans skin lesion may be missed in patients with darker skin color, leading to extracutaneous manifestations in persons with Lyme disease [28]. Despite the mentioned evidence of delayed diagnosis of Lyme disease in patients with SOC, the present literature is deprived of advice about how to accurately diagnose Lyme disease in such patients.

#### Rickettsioses

One article [29] exemplifies the dearth of information regarding the topic at hand and contains the statement that “observed for *Rickettsia rickettsii* rickettsioses, the rash is commonly missed on dark skin,” yet the article does not infer why the rash is missed. While the same article states that “in these cases one should look for scratches or bites instead of the typical rash,” the statement is brief and contains only a few sentences from the entire article. Rickettsioses are also commonly missed in dark skin; for example, 10% of *R rickettsia* cases will not present with a rash, generally in individuals with dark skin or the elderly. In these cases, it is suggested to look for other characteristics such as excoriations [29]. Rocky Mountain spotted fever typically presents with a characteristic petechial rash after 3–5 days, which can be overlooked or missed in dark-skinned individuals/darkly pigmented skin [30–32]. Failure to develop a rash has been associated with increased mortality; however, this may be attributed to neglected, rather than absent, skin findings in dark skin that delay diagnosis and treatment [32, 33]. While physicians should not rely on the cutaneous presentation for diagnosis of rickettsioses, it is an important physical finding for many cases, and when missed in dark skin can lead to delayed diagnosis [31–33].

### Toxin-Mediated Diseases

#### Toxic Shock Syndrome

One co-author of this paper (J. S.) became aware of the Rely tampon/TSS era in the late 1980s and wrote about TSS [34]. During that time period, the association between the use of the highly absorbent Rely tampons and TSS was established and the Rely tampons were removed from the market. Such tampons, being extra-absorbent, were particularly attractive to young women and, therefore, were often left in place for extended periods. A woman who was unfortunate enough to carry a toxin-producing strain of *Staphylococcus aureus* in the vagina was potentially a candidate to develop TSS [35, 36]. In light-skinned women, a faint, sunburn-like rash was often an important early sign of the presence of the TSS, prompting questioning about the possible presence of a tampon; prompt removal of the tampon would reduce the likelihood of the development of full-blown, potentially fatal TSS. Dark-skinned women often did not display the sunburn-like rash, resulting in prolonged tampon use, potentially furthering progression to a septic shock–like syndrome. At present, any young woman developing a progressive septic shock–like syndrome should be asked whether she is menstruating and using a tampon. Prompt tampon removal will reduce the possibility of progressive TSS.

### Fungal Infections

#### Cutaneous Fungal Infections

Dermatophyte infections present uniquely in dark skin as they cause hypopigmentation and cause more pronounced dyspigmentation compared to lighter skin, as the lighter skin tends to cause hyperpigmentation [37–39]. A fungal infection of note is pityriasis versicolor, which presents as hypopigmentation in darker-skinned patients and can manifest in atypical ways [40, 41]. Pityriasis versicolor in dark skin presents with hypopigmentation as well as more facial and neck involvement than other counterparts [42, 43]. While this is a commonly described infection, our search resulted in only 2 included articles on the topic.

## NEXT STEPS

While innovations such as the web application VisualDX have been able to provide more images of dark skin than other resources such as books and DermNet NZ, there are still gaps in the availability of dark skin images [44]. Medical school training materials lack representation of patients with SOC; moreover, 1 study from 2021 found that common medical school resources (including Amboss, Boards and Beyond, Firecracker, First Aid, Pathoma, and the American Academy of Dermatology Basic Dermatology Curriculum) portrayed patients with SOC in only 14.9% of their 1123 image examples [45]. Pathoma contained the most SOC images at 21.4%, exemplifying the limited representation of SOC in medical education materials [45]. To combat the lack of availability of dark skin images, providing a free educational resource rather than a paid subscription service would decrease the financial burden that may deter healthcare professionals from accessing dark skin images. Providing educational modules or electives on the topic for medical students and residents would increase exposure to the diagnoses and lead to recognition of the diseases in the future.

### Limitations

Our search may have been limited by selection bias because we only included research and review articles in English from PubMed or ScienceDirect. Another limitation of this literature review could have been the terms selected for the literature search. We selected broad terms, which possibly excluded more specific articles on the topic. For example, we did not include “African American” as it does not denote actual skin phototype; however, this could lead to exclusion of specific articles.

## CONCLUSIONS

This scoping review identified literature gaps regarding cutaneous manifestations of the infectious diseases HIV, shingles, impetigo, scarlet fever, Lyme disease, TSS, scabies, *Rickettsia*, and cutaneous fungal infections in patients with SOC. It also demonstrated a lack of visual examples and representations of common infectious diseases in such patients. The lack of literature regarding cutaneous manifestations of infectious diseases in patients with SOC may contribute to care barriers in patients with SOC in our healthcare system. Healthcare disparities in infectious diseases and dermatology are clearly present and affect the care of such patients. Increased research is needed in the specialties of both infectious diseases and dermatology. Increased educational collaboration between the 2 specialties will help alleviate the barriers to excellent care faced by patients with SOC.

## Supplementary Material

ofad692_Supplementary_Data

## Supplementary Data

Supplementary materials are available at *Open Forum Infectious Diseases* online. Consisting of data provided by the authors to benefit the reader, the posted materials are not copyedited and are the sole responsibility of the authors, so questions or comments should be addressed to the corresponding author.

## Notes

***Financial support*.** This work was supported by the Infectious Diseases Society of America (G.E.R.M. program grant to D. M.).
