# Supplementary material for: Investigating and Summarizing Information Resources Related to the Clinical Presentation and Diagnosis of Cutaneous Manifestations of Infectious Diseases in Patients With Skin of Color
Source: Open Forum Infect Dis. 2023 Dec 29;11(2):ofad692. doi: 10.1093/ofid/ofad692 (PMC10883730; doi:10.1093/ofid/ofad692)
Supplement: ofad692_Supplementary_Data [file ofad692_supplementary_data.zip › Supplement data.docx]

**Notes:**

Patient consent. The design of this review does not include factors necessitating patient consent.

**Supplemental Data:**

**Pubmed search**

**General search**

"Infectious disease" in patients with "skin of color"

"Infectious disease" in patients with "Dark skin"

"Infectious disease" in patients with "Dark complexion"

"Infectious disease" in patients with "Darkly pigmented skin"

"Infectious disease" in patients with " pigmented skin"

skin manifestations of “infectious disease” in patients with “skin of color”

skin manifestations of “infectious disease” in patients with “pigmented skin”

skin manifestations of infectious disease in “Dark complexion”

skin manifestations of infectious disease in "Dark skin"

skin manifestations of infectious disease in "people of color"

skin manifestations of infectious disease in "Darkly pigmented skin"

cutaneous manifestations of infectious diseases in “skin of color”

cutaneous manifestations of infectious diseases in "pigmented skin"

cutaneous manifestations of infectious diseases in " Darkly pigmented skin"

cutaneous manifestations of infectious diseases in " Dark complexion"

cutaneous manifestations of infectious diseases in " Dark skin"

**Lyme disease**

Lyme disease in “skin of color”

Lyme disease in “pigmented skin”

Lyme disease in " skin of people of color"

Lyme disease in “Dark complexion”

Lyme disease in “Dark skin”

Lyme disease in “Darkly pigmented skin”

Skin manifestations of Lyme disease in “skin of color”

Skin manifestations of Lyme disease in “pigmented skin”

Skin manifestations of Lyme disease in " skin of people of color"

skin manifestations of Lyme Disease in " Dark skin"

skin manifestations of Lyme in " Darkly pigmented skin"

skin manifestations of Lyme in patients with "Dark complexion”

**TSS**

toxic shock syndrome in "skin of color"

toxic shock syndrome in "pigmented skin"

Toxic shock syndrome in "skin of people of color"

Toxic shock syndrome in “Dark complexion”

Toxic shock syndrome in “Dark skin”

Toxic shock syndrome in “Darkly pigmented skin”

Skin Manifestations toxic shock syndrome in "skin of color"

Skin Manifestations toxic shock syndrome in "pigmented skin"

Skin Manifestations Toxic shock syndrome in "skin of people of color"

skin manifestations of Toxic shock syndrome in " Dark skin"

skin manifestations of Toxic shock syndrome in " Darkly pigmented skin"

skin manifestations of Toxic shock syndrome in patients with "Dark complexion”

**HIV**

HIV in "skin of color"

HIV in "pigmented skin"

HIV in "skin of people of color"

HIV in “ Dark complexion”

HIV in “ Dark skin”

HIV in “Darkly pigmented skin”

skin manifestations of HIV in "skin of color"

skin manifestations of HIV in "pigmented skin"

Skin Manifestations HIV in "skin of people of color"

skin manifestations of HIV in " Dark skin"

skin manifestations of HIV in " Darkly pigmented skin"

skin manifestations of HIV in patients with "Dark complexion”

**Scabies**

skin manifestations of Scabies in " Skin of color"

skin manifestations of Scabies in " pigmented skin"

skin manifestations of Scabies in " skin of people of color

skin manifestations of Scabies in " Dark skin"

skin manifestations of Scabies in " Darkly pigmented skin"

skin manifestations of Scabies in patients with "Dark complexion”

Scabies in " Skin of color"

Scabies in "pigmented skin"

Scabies in " skin of people of color"

Scabies in “ Dark complexion”

Scabies in “ Dark skin”

Scabies in “Darkly pigmented skin”

**Shingles**

Shingles in " skin of people of color"

Shingles in “ skin of color”

Shingles in “ pigmented skin”

herpes zoster in " skin of color"

Herpes zoster in “ Skin of people of color”

herpes zoster in "pigmented skin"

Shingles in “ Dark complexion”

Shingles in “ Dark skin”

Shingles in “Darkly pigmented skin”

Herpes Zoster in “ Dark complexion”

Herpes Zoster in “ Dark skin”

Herpes Zoster in “Darkly pigmented skin”

Skin manifestations of Shingles in " skin of people of color"

Skin manifestations Shingles in “ skin of color”

Skin manifestations Shingles in “ pigmented skin”

Skin manifestations herpes zoster in " skin of color"

Skin manifestations Herpes zoster in “ Skin of people of color”

Skin manifestations herpes zoster in "pigmented skin"

Skin manifestations of Shingles in “ Dark complexion”

Skin manifestations of Shingles in “ Dark skin”

Skin manifestations of Shingles in “Darkly pigmented skin”

Skin manifestations of Herpes Zoster in “ Dark complexion”

Skin manifestations of Herpes Zoster in “ Dark skin”

Skin manifestations of Herpes Zoster in “Darkly pigmented skin”

**Impetigo**

impetigo in “skin of color”

impetigo in "pigmented skin"

impetigo in "skin of people of color"

impetigo in “ Dark complexion”

impetigo in “ Dark skin”

impetigo in “Darkly pigmented skin”

skin manifestation of impetigo in “skin of color”

Skin manifestations of impetigo in “ pigmented skin”

Skin manifestations of impetigo in “ Skin of people of color”

Skin manifestations of impetigo in “ Dark complexion”

Skin manifestations of impetigo in “ Dark skin”

Skin manifestations of impetigo in “ Darkly Pigmented skin”

**Scarlet fever**

Scarlet fever in “skin of color”

Scarlet fever in "pigmented skin"

Scarlet fever in “ Skin of people of color”

Scarlet fever in “ Dark complexion”

Scarlet fever “ Dark skin”

Scarlet fever “Darkly pigmented skin”

skin manifestations scarlet fever in “skin of color”

skin manifestations scarlet fever in "pigmented skin"

skin manifestations scarlet fever in “skin of people of color”

Skin manifestations of Scarlet fever in “ Dark complexion”

Skin manifestations of Scarlet fever in “ Dark skin”

Skin manifestations of Scarlet fever in “ Darkly Pigmented skin”

**Fungal infections**

Cutaneous fungal infection in “skin of color”

Cutaneous fungal infection in “pigmented skin”

Cutaneous fungal infection in "skin of people of color"

Cutaneous fungal infection in “Dark complexion”

Cutaneous fungal infection in “Dark skin”

Cutaneous fungal infection in “Darkly pigmented skin”

Skin manifestations of fungal infection in “skin of color”

Skin manifestations of fungal infection in “pigmented skin”

Skin manifestations of fungal infection in "skin of people of color"

Skin manifestations of fungal infection in "Darkly pigmented skin"

Skin manifestations of fungal infection in “Dark skin”

Skin manifestations of fungal infection in “Dark complexion”

**Rickettsia**

Rickettsia in “skin of color”

Rickettsia in “pigmented skin”

Rickettsia in "skin of people of color"

Rickettsia in “Dark Complexion”

Rickettsia in “Dark skin”

Rickettsia in “Darkly pigmented skin”

Skin manifestations of Rickettsia in “skin of color”

Skin manifestations of Rickettsia in “pigmented skin”

Skin manifestations of Rickettsia in "skin of people of color"

Skin manifestations of Rickettsia in "Darkly pigmented skin"

Skin manifestations of Rickettsia in “Dark skin”

Skin manifestations of Rickettsia in “Dark complexion”

**Science Direct search**

"Infectious disease" in patients with "skin of color

skin manifestations of “infectious disease” in patients with “skin of color”

skin manifestations of “infectious disease” in patients with “pigmented skin”

skin manifestations of “infectious disease” in “dark complexion”

skin manifestations of infectious disease in "people of color"

skin manifestations of infectious disease in “ Darkly pigmented skin”

skin manifestations of infectious disease in “dark skin”

"Infectious disease" in patients with "Dark skin"

"Infectious disease" in patients with "Dark complexion"

"Infectious disease" in patients with "Darkly pigmented skin"

**Lyme disease**

Lyme disease in “skin of color”

Lyme disease in “pigmented skin”

Lyme disease in " skin of people of color"

Lyme disease in “Dark complexion”

Lyme disease in “Dark skin”

Lyme disease in “Darkly pigmented skin”

Skin manifestations of Lyme disease in “skin of color”

Skin manifestations of Lyme disease in “pigmented skin”

Skin manifestations of Lyme disease in " skin of people of color"

Skin manifestations of Lyme disease in "Darkly pigmented skin"

Skin manifestations of Lyme disease in “Dark skin”

Skin manifestations of Lyme disease in “Dark complexion”

**Toxic shock syndrome**

toxic shock syndrome in "skin of color"

toxic shock syndrome in "pigmented skin"

Toxic shock syndrome in "skin of people of color"

Toxic shock syndrome in “Dark complexion”

Toxic shock syndrome in “Dark skin”

Toxic shock syndrome in “Darkly pigmented skin”

Skin Manifestations of toxic shock syndrome in "skin of color"

Skin Manifestations of toxic shock syndrome in "pigmented skin"

Skin Manifestations of Toxic shock syndrome in "skin of people of color"

Skin manifestations of Toxic shock syndrome in "Darkly pigmented skin"

Skin manifestations of Toxic shock syndrome in “Dark skin”

Skin manifestations of Toxic shock syndrome in “Dark complexion”

**HIV**

HIV in "skin of color"

HIV in "pigmented skin"

HIV in "skin of people of color"

HIV in “Dark complexion”

HIV in “Dark skin”

HIV in “Darkly pigmented skin”

skin manifestations of HIV in "skin of color"

skin manifestations of HIV in "pigmented skin"

Skin Manifestations HIV in "skin of people of color"

Skin manifestations of HIV in "Darkly pigmented skin"

Skin manifestations of HIV in “Dark skin”

Skin manifestations of HIV in “Dark complexion”

**Scabies**

skin manifestations of Scabies in " Skin of color"

skin manifestations of Scabies in " pigmented skin"

skin manifestations of Scabies in " skin of people of color

Scabies in " Skin of color"

Scabies in "pigmented skin"

Scabies in " skin of people of color"

Scabies in “Dark complexion”

Scabies in “Dark skin”

Scabies in “Darkly pigmented skin”

Skin manifestations of Scabies in "Darkly pigmented skin"

Skin manifestations of Scabies in “Dark skin”

Skin manifestations of Scabies in “Dark complexion”

**Shingles**

Shingles in " skin of people of color"

Shingles in “ skin of color”

Shingles in “ pigmented skin”

Shingles in “ Dark complexion”

Shingles in “ Dark skin”

Shingles in “Darkly pigmented skin”

herpes zoster in " skin of color"

Herpes zoster in “ Skin of people of color”

herpes zoster in "pigmented skin"

Skin manifestations of Shingles in " skin of people of color"

Skin manifestations Shingles in “ skin of color”

Skin manifestations Shingles in “ pigmented skin”

Skin manifestations of Shingles in "Darkly pigmented skin"

Skin manifestations of Shingles in “Dark skin”

Skin manifestations of Shingles in “Dark complexion”

Skin manifestations herpes zoster in " skin of color"

Skin manifestations Herpes zoster in “ Skin of people of color”

Skin manifestations herpes zoster in "pigmented skin"

Skin manifestations herpes zoster in "Darkly pigmented skin"

Skin manifestations herpes zoster in "Dark skin”

Skin manifestations herpes zoster in "Dark complexion”

**Impetigo**

impetigo in “skin of color”

impetigo in "pigmented skin"

impetigo in "skin of people of color"

impetigo in “ Dark complexion”

impetigo in “ Dark skin”

impetigo in “Darkly pigmented skin”

skin manifestation of impetigo in “skin of color”

Skin manifestations of impetigo in “ pigmented skin”

Skin manifestations in of impetigo “ Skin of people of color”

Skin manifestations of impetigo in “ Dark complexion”

Skin manifestations of impetigo in “ Dark skin”

Skin manifestations of impetigo in “ Darkly Pigmented skin” .

**Scarlet fever**

Scarlet fever in “skin of color”

Scarlet fever in "pigmented skin"

Scarlet fever in “ Skin of people of color”

Scarlet fever in “ Dark complexion”

Scarlet fever “ Dark skin”

Scarlet fever “Darkly pigmented skin”

skin manifestations of scarlet fever in “skin of color”

skin manifestations of scarlet fever in "pigmented skin"

skin manifestations of scarlet fever in “skin of people of color”

Skin manifestations of Scarlet fever in “ Dark complexion”

Skin manifestations of Scarlet fever in “ Dark skin”

Skin manifestations of Scarlet fever in “ Darkly Pigmented skin”

**Fungal infections**

Cutaneous fungal infection in “skin of color”

Cutaneous fungal infection in “pigmented skin”

Cutaneous fungal infection in "skin of people of color"

Cutaneous fungal infection in “Dark complexion”

Cutaneous fungal infection in “Dark skin”

Cutaneous fungal infection in “Darkly pigmented skin”

Skin manifestations of fungal infection in “skin of color”

Skin manifestations of fungal infection in “pigmented skin”

Skin manifestations of fungal infection in "skin of people of color"

Skin manifestations of fungal infection in "Darkly pigmented skin"

Skin manifestations of fungal infection in “Dark skin”

Skin manifestations of fungal infection in “Dark complexion”

**Rickettsia**

Rickettsia in “skin of color”

Rickettsia in “pigmented skin”

Rickettsia in "skin of people of color"

Rickettsia in “Dark Complexion”

Rickettsia in “Dark skin”

Rickettsia in “Darkly pigmented skin”

Skin manifestations of Rickettsia in “skin of color”

Skin manifestations of Rickettsia in “pigmented skin”

Skin manifestations of Rickettsia in "skin of people of color"

Skin manifestations of Rickettsia in "Darkly pigmented skin"

Skin manifestations of Rickettsia in “Dark skin”

Skin manifestations of Rickettsia in “Dark complexion”

**PRISMA Checklist**

**Preferred Reporting Items for Systematic reviews and Meta-Analyses extension for Scoping Reviews (PRISMA-ScR) Checklist**

| **SECTION** | **ITEM** | **PRISMA-ScR CHECKLIST ITEM** | **REPORTED ON PAGE #** |
| --- | --- | --- | --- |
| **TITLE** | | | |
| Title | 1 | Identify the report as a scoping review. | Click here to enter text. |
| **ABSTRACT** | | | |
| Structured summary | 2 | Provide a structured summary that includes (as applicable): background, objectives, eligibility criteria, sources of evidence, charting methods, results, and conclusions that relate to the review questions and objectives. | Click here to enter text. |
| **INTRODUCTION** | | | |
| Rationale | 3 | Describe the rationale for the review in the context of what is already known. Explain why the review questions/objectives lend themselves to a scoping review approach. | Click here to enter text. |
| Objectives | 4 | Provide an explicit statement of the questions and objectives being addressed with reference to their key elements (e.g., population or participants, concepts, and context) or other relevant key elements used to conceptualize the review questions and/or objectives. | Click here to enter text. |
| **METHODS** | | | |
| Protocol and registration | 5 | Indicate whether a review protocol exists; state if and where it can be accessed (e.g., a Web address); and if available, provide registration information, including the registration number. | Click here to enter text. |
| Eligibility criteria | 6 | Specify characteristics of the sources of evidence used as eligibility criteria (e.g., years considered, language, and publication status), and provide a rationale. | Click here to enter text. |
| Information sources* | 7 | Describe all information sources in the search (e.g., databases with dates of coverage and contact with authors to identify additional sources), as well as the date the most recent search was executed. | Click here to enter text. |
| Search | 8 | Present the full electronic search strategy for at least 1 database, including any limits used, such that it could be repeated. | Click here to enter text. |
| Selection of sources of evidence† | 9 | State the process for selecting sources of evidence (i.e., screening and eligibility) included in the scoping review. | Click here to enter text. |
| Data charting process‡ | 10 | Describe the methods of charting data from the included sources of evidence (e.g., calibrated forms or forms that have been tested by the team before their use, and whether data charting was done independently or in duplicate) and any processes for obtaining and confirming data from investigators. | Click here to enter text. |
| Data items | 11 | List and define all variables for which data were sought and any assumptions and simplifications made. | Click here to enter text. |
| Critical appraisal of individual sources of evidence§ | 12 | If done, provide a rationale for conducting a critical appraisal of included sources of evidence; describe the methods used and how this information was used in any data synthesis (if appropriate). | Click here to enter text. |
| Synthesis of results | 13 | Describe the methods of handling and summarizing the data that were charted. | Click here to enter text. |
| **RESULTS** | | | |
| Selection of sources of evidence | 14 | Give numbers of sources of evidence screened, assessed for eligibility, and included in the review, with reasons for exclusions at each stage, ideally using a flow diagram. | Click here to enter text. |
| Characteristics of sources of evidence | 15 | For each source of evidence, present characteristics for which data were charted and provide the citations. | Click here to enter text. |
| Critical appraisal within sources of evidence | 16 | If done, present data on critical appraisal of included sources of evidence (see item 12). | Click here to enter text. |
| Results of individual sources of evidence | 17 | For each included source of evidence, present the relevant data that were charted that relate to the review questions and objectives. | Click here to enter text. |
| Synthesis of results | 18 | Summarize and/or present the charting results as they relate to the review questions and objectives. | Click here to enter text. |
| **DISCUSSION** | | | |
| Summary of evidence | 19 | Summarize the main results (including an overview of concepts, themes, and types of evidence available), link to the review questions and objectives, and consider the relevance to key groups. | Click here to enter text. |
| Limitations | 20 | Discuss the limitations of the scoping review process. | Click here to enter text. |
| Conclusions | 21 | Provide a general interpretation of the results with respect to the review questions and objectives, as well as potential implications and/or next steps. | Click here to enter text. |
| **FUNDING** | | | |
| Funding | 22 | Describe sources of funding for the included sources of evidence, as well as sources of funding for the scoping review. Describe the role of the funders of the scoping review. | Click here to enter text. |

JBI = Joanna Briggs Institute; PRISMA-ScR = Preferred Reporting Items for Systematic reviews and Meta-Analyses extension for Scoping Reviews.

* Where *sources of evidence* (see second footnote) are compiled from, such as bibliographic databases, social media platforms, and Web sites.

† A more inclusive/heterogeneous term used to account for the different types of evidence or data sources (e.g., quantitative and/or qualitative research, expert opinion, and policy documents) that may be eligible in a scoping review as opposed to only studies. This is not to be confused with *information sources* (see first footnote).

‡ The frameworks by Arksey and O’Malley (6) and Levac and colleagues (7) and the JBI guidance (4, 5) refer to the process of data extraction in a scoping review as data charting*.*

§ The process of systematically examining research evidence to assess its validity, results, and relevance before using it to inform a decision. This term is used for items 12 and 19 instead of "risk of bias" (which is more applicable to systematic reviews of interventions) to include and acknowledge the various sources of evidence that may be used in a scoping review (e.g., quantitative and/or qualitative research, expert opinion, and policy document).

*From:* Tricco AC, Lillie E, Zarin W, O'Brien KK, Colquhoun H, Levac D, et al. PRISMA Extension for Scoping Reviews (PRISMAScR): Checklist and Explanation. Ann Intern Med. 2018;169:467–473. [doi: 10.7326/M18-0850](http://annals.org/aim/fullarticle/2700389/prisma-extension-scoping-reviews-prisma-scr-checklist-explanation).
