# Supplementary figures and images for: Investigating and Summarizing Information Resources Related to the Clinical Presentation and Diagnosis of Cutaneous Manifestations of Infectious Diseases in Patients With Skin of Color
Source: Open Forum Infect Dis. 2023 Dec 29;11(2):ofad692. doi: 10.1093/ofid/ofad692 (PMC10883730; doi:10.1093/ofid/ofad692)

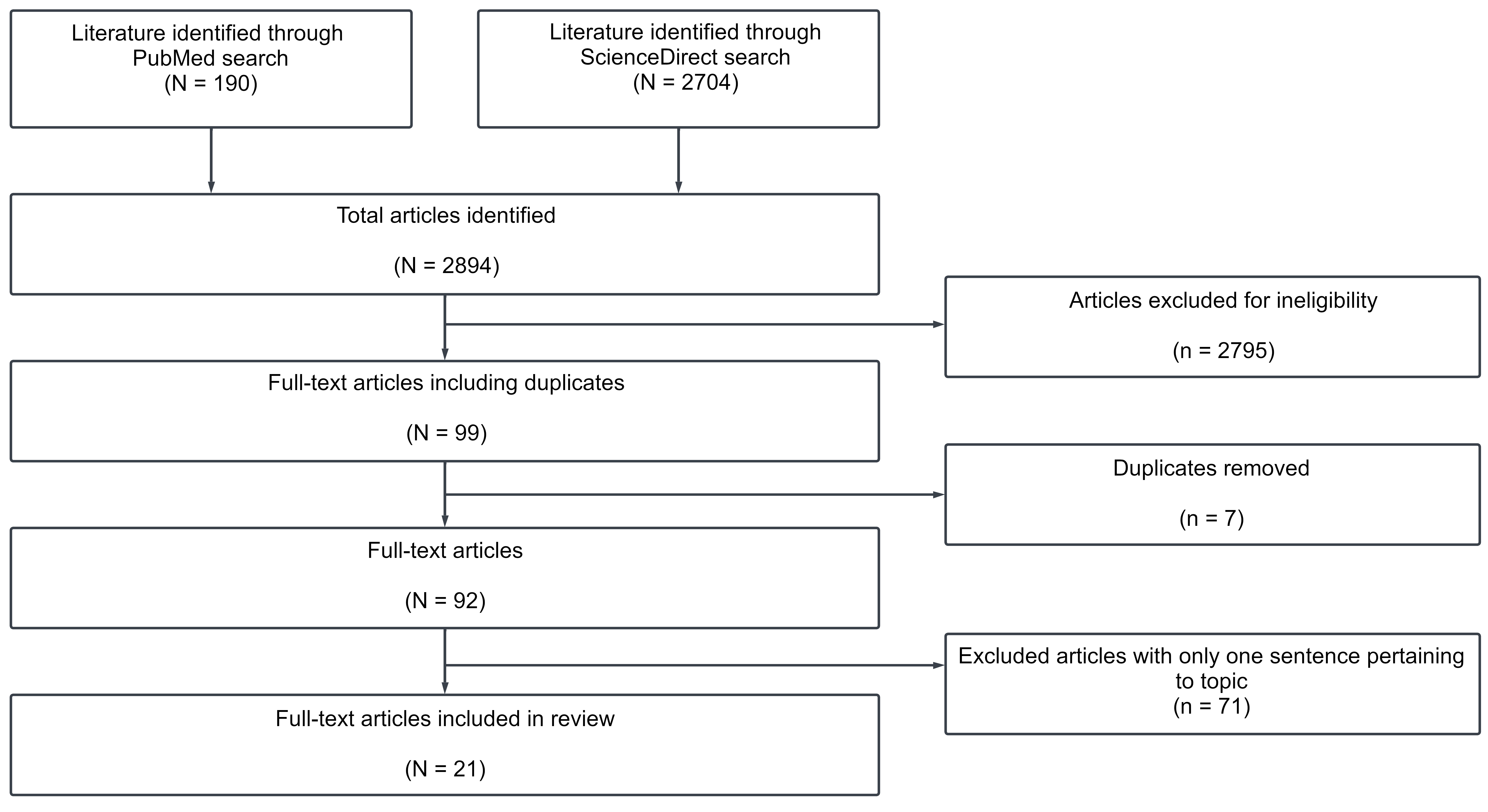

Supplement: ofad692_Supplementary_Data [file ofad692_supplementary_data.zip › figure 1.tiff.tif]
